# Gemcitabine sensitivity-related mRNA expression in endoscopic ultrasound-guided fine-needle aspiration biopsy of unresectable pancreatic cancer

**DOI:** 10.1186/1756-9966-28-83

**Published:** 2009-06-16

**Authors:** Reiko Ashida, Bunzo Nakata, Minoru Shigekawa, Nobumasa Mizuno, Akira Sawaki, Kosei Hirakawa, Tetsuo Arakawa, Kenji Yamao

**Affiliations:** 1 Department of Gastroenterology, Osaka City University Graduate School of Medicine, Osaka, Japan; 2 Department of Surgical Oncology, Osaka City University Graduate School of Medicine, Osaka, Japan; 3 Department of Gastroenterology, Aichi Cancer Center Hospital, Nagoya, Japan

## Abstract

**Background:**

The aim of this study was to determine a predictive indicator of gemcitabine (GEM) efficacy in unresectable pancreatic cancer using tissue obtained by endoscopic ultrasound-guided fine-needle aspiration biopsy (EUS-FNA).

**Methods:**

mRNAs extracted from 35 pancreatic tubular adenocarcinoma tissues obtained by EUS-FNA before GEM-treatment were studied. mRNAs were amplified and applied to a Focused DNA Array, which was restricted to well-known genes, including GEM sensitivity-related genes, deoxycytidine kinase (dCK), human equilibrative nucleoside transporter 1 (hENT1), hENT2, dCMP deaminase, cytidine deaminase, 5'-nucleotidase, ribonucleotide reductase 1 (RRM1) and RRM2. mRNA levels were classified into high and low expression based on a cut-off value defined as the average expression of 35 samples. These 35 patients were divided into the following two groups. Patients with partial response and those with stable disease whose tumor markers decreased by 50% or more were classified as the effective group. The rest of patients were classified as the non-effective group. The relationship between GEM efficacy and mRNA expression was then examined by chi-squared test.

**Results:**

Among these GEM sensitivity-related genes, dCK alone showed a significant correlation with GEM efficacy. Eight of 12 patients in the effective group had high dCK expression, whereas 16 of 23 patients in non-effective group had low dCK expressions (*P *= 0.0398).

**Conclusion:**

dCK mRNA expression is a candidate indicator for GEM efficacy in unresectable pancreatic cancer. Quantitative mRNA measurements of dCK using EUS-FNA samples are necessary for definitive conclusions.

## Background

Pancreatic cancer is one of the most lethal human cancers. The standard treatment for unresectable pancreatic cancer was previously 5-fluorouracil (5-FU)-based chemotherapy. In 1997, however, it was reported that gemcitabine (GEM) conferred significantly longer survival and clinical benefits when compared to 5-FU in patients with locally advanced or metastatic pancreatic cancer [1]. Since that time, GEM has been recognized as the standard treatment for this disease.

Recent investigations using cell lines or surgical specimens have revealed that the expressions of human equilibrative nucleoside transporter 1 (hENT1) [2-4] and the GEM-metabolism-related enzymes such as deoxycytidine kinase (dCK) [5,6] are putative predictors for the efficacy of GEM treatment. If GEM could be selectively administered to patients with GEM-sensitive tumors based on the expression of these genes in the tumor, maximum efficacy could be achieved and the unpleasant side effects in GEM-resistant patients may be avoided.

Focused DNA array (FDA), a DNA microarray restricted to tens to hundreds of well-known genes, is an ideal tool for comprehensive analysis of GEM sensitivity-related genes, as it has the ability to simultaneous measure the expression of a number of genes. DNA microarray analysis has rarely been used to study unresectable pancreatic cancer, because obtaining pancreatic cancer cells without surgery has traditionally been difficult. However, the recent development of endoscopic ultrasound-guided fine-needle aspiration (EUS-FNA) has allowed pancreatic tissue to be obtained safely. This technique thus opens new possibilities for the diagnosis of pancreatic cancer, not only by pathology, but also by gene analysis, such as for the K-*ras *mutation [7,8]. The aim of this study was to identify possible predictors of GEM efficacy using EUS-FNA samples of unresectable pancreatic cancer by means of FDA analysis.

## Methods

### EUS-FNA procedure

Thirty-five patients with unresectable pancreatic ductal cancers treated with GEM were studied. EUS-FNA was performed before GEM-treatment and the procedures were as described elsewhere [9]. In brief, the lesion was identified on B-mode imaging. The absence of vessels in the target area was confirmed with the color Doppler mode. After determination of the adequate angle to the tumor, an aspiration needle was introduced into the lesion. While the catheter connected to the needle was sucked by a 20 ml syringe, the needle was moved back and forth 20–30 times within the tumor. The negative pressure was released before the needle was removed from the lesion. To obtain sufficient tissue for RNA extraction and pathological diagnosis, several biopsy specimens were collected from each tumor by EUS-FNA using 19 or 22-gauge aspiration needles (ECHOTIP ULTRA; Wilson-Cook Medical Inc., Winston-Salem, NC, USA). A 19-gauge needle can take more amount of specimen than a 22-gauge needle. However, a 22-gauge needle gives less damage to tissue than a 19-gauge needle and can take enough specimen for the diagnosis and the analysis. We used 19-gauge needles for the first nine cases. For the following 26 cases, the tissues were obtained by 22-gauge needles. A cytopathologist immediately examined the specimens for cancer cells using part of the obtained tissue.

### RNA extraction

To ensure RNA quality, the obtained tissue was instantly immersed in 1 ml of RNAlater (Ambion, Austin, TX, USA) and incubated overnight in reagent at 4°C. Tissue samples were then removed from RNAlater and transferred to -80°C for storage. Total RNAs were extracted using the RNeasy Mini Kit (Qiagen GmbH, Hilden, Germany) according to the manufacturer's instructions. Amounts of RNA were measured using a NanoDrop ND-1000 Spectrophotometer (NanoDrop Technologies, Inc., Wilmington, DE, USA). RNAs were examined for qualities by confirming the 28S and 18S ribosomal bands with an Agilent 2100 Bioanalyzer (Agilent Technologies, Inc., Santa Clara, CA, USA). RNA samples were subjected to FDA analyses after amplification.

#### Patients

The patients with advanced pancreatic cancer, who were admitted to Aichi Cancer Center Hospital from November, 2004 to April, 2007 and were planed to treat with GEM monotherapy, were consecutively entered into this study. GEM monotherapy was performed for all patients by administering intravenous GEM at a dose of 1000 mg/m^2 ^for 30 minutes on days 1, 8 and 15 of a 28-day cycle. The patients were assessed for definitive GEM efficacy, and were thus investigated for correlations between GEM sensitivity-related gene expression and clinical efficacy of GEM monotherapy. Clinicopahtologic data for the 35 patients are shown in Table 1. Evaluation of response to GEM by imaging study was based on the Response Evaluation Criteria in Solid Tumors (RECIST). The GEM-effective patients were defined as having a partial response (PR) by imaging studies or as having stable disease (SD) by imaging studies and a 50% or more decrease in both of abnormal CA 19-9 and CEA titers in sera, as compared to pretreatment values.

**Table 1:** Clinical characteristics of patients receiving GEM monotherapy.

Number of patients		35
Age (y)	Mean ± SD (Range)	61.3 ± 8.5 (46–77)
Gender	Male:Female	16: 19
Location	Head: Body/tail	7: 28
Follow-up time from commencement of GEM monotherapy (mo)		
	Median (Range)	7.7 (3.0–21.4)
Number of courses of GEM monotherapy		
	Mean ± SD (Range)	5.9 ± 4.0 (2–16)
GEM efficacy	Effective*: Non-effective	12: 23

GEM, gemcitabine

*Effective, partial response by imaging study or stable disease by imaging study with 50% or more decrease in tumor markers compared to pretreatment values

This study was performed in accordance with the human and ethical principles of research set forth in the Helsinki guidelines. Informed consent was obtained from all patients who participated in the investigation. This study was approved by the institutional review boards of Osaka City University Graduate School of Medicine and Aichi Cancer Center.

#### RNA isolation linear RNA polymerase amplification

The extracted RNA from EUS-FNA sample was insufficient for FDA analysis; therefore, RNA were amplified as described elsewhere [10]. Briefly, the sample RNA was subjected to reverse transcription with T7 RNA polymerase-based linear amplification using the Agilent Low RNA Input Linear Amplification Kit (Agilent Technology, Inc.) to synthesize cDNA. The same kit was used for synthesized cDNA to amplify antisense RNA (aRNA) by *in vitro *transcription using T7 RNA polymerase. During this procedure, amplified aRNAs from the sample and the reference RNA (mix of RNAs from pancreatic cancer cell line BxPC-3 and colon cancer cell line DLD-1, 1:1 ratio) were labeled with Cyanine 5 (cy5) and Cyanine 3 (cy3) monofunctional reactive dyes (GE Healthcare Bio-Sciences AB, Uppsala, Sweden), respectively.

#### FDA analysis

FDA included 133 genes that code sensitivity-related factors such as thymidylate synthase (TS) and dihydropyrimidine dehydrogenase (DPD), and molecular targets such as epidermal growth factor receptor (EGFR) and vascular endothelial growth factor (VEGF). With regard to GEM sensitivity-related factors, dCK, hENT1, hENT2, deoxycytidylate deaminase (DCD), cytidine deaminase (CDA), 5'-nucleotidase (5'-NT), ribonucleotide reductase 1 (RRM1) and RRM2 were included on FDA.

**Figure 1:**
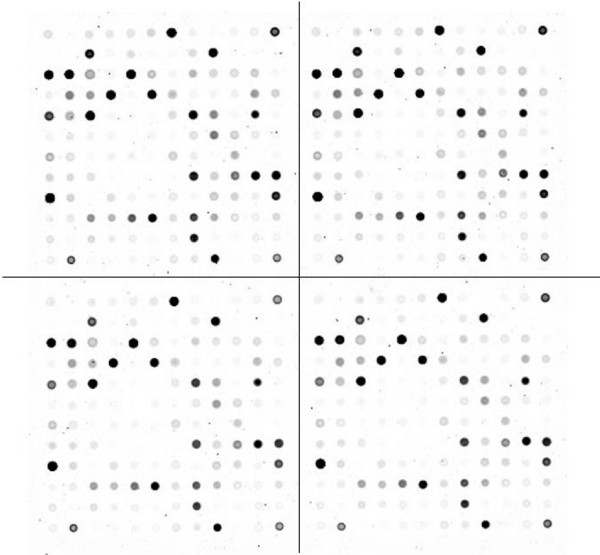
**Focused DNA array containing quadruplicate sets of oligonucleotide array sequences for 133 genes**. High reproducibility of gene expression is confirmed in corresponding spots of the quadruplicate.

Fluorescent aRNAs obtained from 35 RNA samples were provided for FDA hybridization according to Agilent's oligonucleotide microarray hybridization user's manual. The hybridized FDA was scanned with an Agilent dual-laser DNA microarray scanner G2565AA. Feature extraction and data normalization were conducted with Agilent Feature Extraction software. Relative expression levels were measured by normalizing the signal intensities of Cy5 to those of Cy3. The mean of four replicate samples was used for each experiment (Fig. 1). Data were expressed as relative values against a house-keeping gene, glyceraldehyde 3-phosphate dehydrogenase (GAPDH).

### Statistical analysis

High mRNA expression was defined as above the average value of the 35 RNA samples. The relationship between mRNA expression and GEM efficacy was examined by chi-squared test (Fisher's exact test). Survival data were estimated by the Kaplan-Meier method and were examined by log-rank test.

## Results

### Clinical outcome

**Figure 2:**
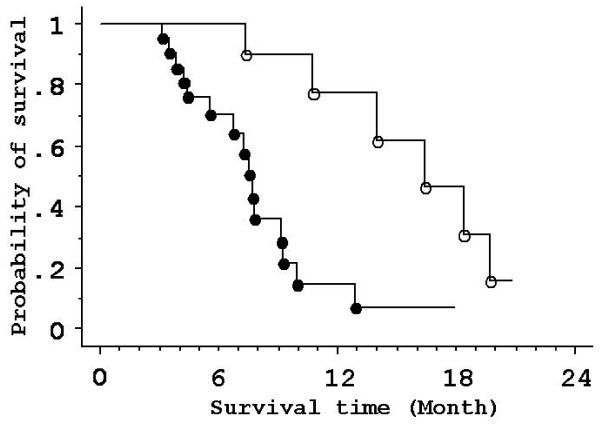
**Probability of survival for patients with unresectable pancreatic ductal cancer stratified by gemcitabine efficacy**. Open circles, GEM-effective group. Closed circles, GEM-non-effective group. There is a significant difference between survival in the two groups.

Five of 35 patients who completed two courses of GEM monotherapy showed PR, SD was seen in 19 patients, and progressive disease was seen in 11 patients. Among the 19 SD patients, pretreatment values for tumor markers in two patients were normal. Abnormal levels of tumor markers in seven of 17 SD-patients decreased by 50% or more as compared to pretreatment values. When GEM efficacy was defined as PR or SD with a 50% or more decrease in tumor markers compared to baseline, 12 patients were classified into the effective group (Table 1). There was a significant difference between the survival periods of the effective and the non-effective groups (Median survival time, 16.6 months vs. 7.8 months, respectively; *P *= 0.0017) (Fig. 2).

### RNA quantity and quality

**Figure 3:**
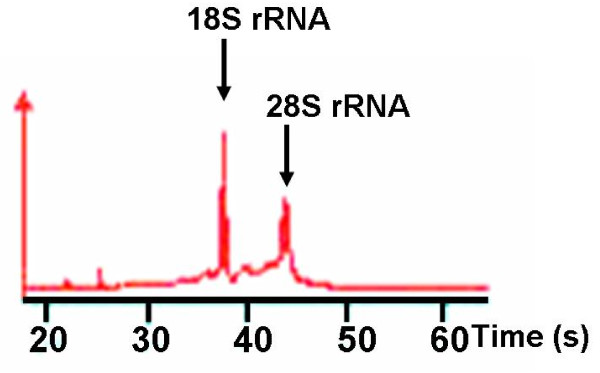
**Representative electropherogram of total RNA extracted from pancreatic cancer obtained by endoscopic ultrasound-guided fine-needle aspiration biopsy**. The ratio of 28S to 18S of ribosomal RNA indicates good quality of total RNA.

Mean ± SD amount of total RNA from 35 tumors was 0.7 ± 0.7 μg (range, 0.1 – 3.0 μg). All 35 RNA samples were of sufficient quality (Fig. 3).

### GEM sensitivity-related gene expression and clinical GEM efficacy

Gene expressions as relative values against GAPDH were as follows: hENT-1, 3.88 (mean), 2.77–6.41 (range); hENT-2, 4.04, 2.54–6.68; dCK, 3.90, 2.21–6.79; DCD, 4.61, 3.09–7.60; CDA, 2.71, 0.27–7.89; 5'-NT, 4.30, 1.35–7.23; RRM1, 2.02, 0.41–5.53; RRM2, 0.91, 0.18–3.34. Among GEM sensitivity-related genes, dCK mRNA expression alone predicted GEM efficacy (Table 2). Eight of 12 tumors in the effective group had high dCK expression while 16 of 23 tumors in the non-effective group had low dCK expression (*P *= 0.0398).

**Table 2:** Correlation between gene expression and GEM efficacy in patients with pancreatic cancer receiving GEM monotherapy.

		GEM efficacy		
			
Gene Expression*		Effective^§^	Non-effective	*P*^¶^-value
hENT1	High	4	9	>0.9999
	Low	8	14	
hENT2	High	6	9	0.5374
	Low	6	14	
dCK	High	8	7	0.0398
	Low	4	16	
DCD	High	3	9	0.4765
	Low	9	14	
CDA	High	4	9	>0.9999
	Low	8	14	
5'-NT	High	4	12	0.2882
	Low	8	11	
RRM1	High	4	8	>0.9999
	Low	8	15	
RRM2	High	4	8	>0.9999
	Low	8	15	

GEM, gemcitabine

*Gene expression was determined as high or low based on mean values of 35 EUS-FNA samples.

^§^Effective, partial response by imaging study or stable disease by imaging study with 50% or more decrease in tumor markers compared to pretreatment value

^¶^*P*, examined by chi-squared test (Fisher's exact test)

## Discussion

EUS-FNA is widely used as a cytological and histological diagnostic method for pancreatic cancer [8,11]. However, there have been few reports on gene analysis of pancreatic cancer using EUS-FNA samples [7,8,12]. In contrast, a number of studies have demonstrated the feasibility of DNA microarray analysis using samples obtained by FNA in other malignancies, such as breast cancer and lung cancer [13-15]. At least 10 μg of total RNA is required for DNA microarray analysis [10]. Due to the low volume of biopsy specimens obtained by EUS-FNA, it is typically impossible to perform DNA microarray analysis using the raw RNA extracted from these samples. However, a high-fidelity RNA amplification protocol has recently been established [10,16] that allows analysis of gene expression profiles using small volumes RNA, such as those obtained by EUS-FNA. In our series, only 0.1 – 3.0 μg of total RNA was extracted from EUS-FNA biopsy samples.

The objective response rate of GEM monotherapy for pancreatic cancer has been reported to be 5–12% [1,17,18]. In this study, PR was observed in 5 of 35 (14%) patients treated with GEM monotherapy, which corresponds with the response rates reported previously. The number of patients in the GEM-effective group was too small to evaluate for correlations between GEM efficacy and mRNA expression. Therefore, SD patients with a 50% or more decrease in abnormal serum levels of tumor markers compared to baseline were included in the GEM-effective group. CA 19-9 has been shown to be correlated with clinical efficacy of GEM in pancreatic cancer [19]. In this study, the GEM-effective group had a significantly better prognosis than the non-effective group, indicating that the grouping based on GEM efficacy was appropriate.

GEM is transported into the cell largely via hENT1 and partly via hENT2 [4]. It has been reported that pancreatic cancer patients with high hENT1 protein [2] or mRNA [3] expression in surgical specimens or biopsies have significantly longer survival after GEM treatment, as compared to those with low levels of hENT1. In *in vitro *experiments, high hENT1 mRNA levels have been shown to be associated with GEM sensitivity, as represented by IC_50 _values [20,21]. In cells, GEM is phosphorylated to its active metabolites by dCK. Several reports have suggested that high dCK enzyme activity may contribute to GEM sensitivity in experimental settings [5] and surgical samples [6]. However, GEM is inactivated by deamination, as catalyzed by DCD. CDA and 5'-NT are also a catabolic enzymes of GEM. Therefore, resistance to GEM may be induced by increased activity of DCD, CDA or 5'-NT [3,5,22]. Ribonucleotide reductase, which consists of dimerized large and small RRM1 and RRM2 subunits, is the rate-limiting enzyme for DNA synthesis, as it is the only known enzyme that converts ribonucleotides to deoxyribonucleotides. GEM exerts its cytotoxicity by inhibiting ribonucleotide reductase. High expression of RRM1 and RRM2 has been suggested to be a mechanism of GEM resistance [22-26]. Thus, several metabolic enzymes and nucleoside transporters have been suggested to affect GEM sensitivity. FDA analysis may therefore be suitable to identify predictors of GEM efficacy by using a very small quantity of samples taken by EUS-FNA from unresectable pancreatic cancer, as it can simultaneously assess the expression of multiple mRNAs related to GEM sensitivity. Our results suggested that high dCK mRNA expression is a predictor of GEM efficacy. In these experimental settings, RNA from most samples were subjected to FDA analysis and were not subjected to further assessment. However, to confirm the relationship between dCK mRNA expression and GEM efficacy, quantitative measurement of expression by real-time reverse transcription-polymerase chain reaction is required. In this study, other GEM sensitivity-related gene expressions including hENT-1 could not be proved to be predictors for GEM efficacy. However, these gene expressions may not be totally denied as predictors of GEM efficacy by the present study using small number of samples. The contamination of normal tissue into tumor tissue obtained by EUS-FNA may also be a major obstacle to an accurate analysis. Microdissection technique for EUS-FNA sample might be required to avoid the normal tissue contamination.

## Conclusion

In conclusion, dCK mRNA expression in EUS-FNA biopsy specimens may be a predictor for response to GEM in patients with unresectable pancreatic cancer. The FDA used in this study also contained molecular target genes that may be promising for the treatment of pancreatic cancer. These data may be helpful for future cancer treatments that target specific molecules.

## List of abbreviations

GEM: gemcitabine; EUS-FNA: endoscopic ultrasound-guided fine-needle aspiration biopsy; dCK: deoxycytidine kinase; hENT1: human equilibrative nucleoside transporter 1; RRM1: ribonucleotide reductase 1; 5-FU: 5-fluorouracil; FDA: Focused DNA array; PR: partial response; SD: stable disease; aRNA: antisense RNA: cy5: cyanine 5; cy3: cyanine 3; TS: thymidylate synthase; DPD: dihydropyrimidine dehydrogenase; EGFR: epidermal growth factor receptor; VEGF: vascular endothelial growth; DCD: deoxycytidylate deaminase; CDA: cytidine deaminase; 5'-NT: 5'-nucleotidase; GAPDH: glyceraldehyde 3-phosphate dehydrogenase.

## Competing interests

The authors declare that they have no competing interests.

## Authors' contributions

RA and BN have made substantial contributions to conception, design, data analysis, interpretation of data, and drafting the manuscript. MS, NM, AS, and KY have made substantial contributions to patients sample collection and acquisition of data. KH and TA have made contributions to revising the manuscript critically for important intellectual content. All authors read and approved the final manuscript.
